# Polarized benzene rings can promote the interaction between CaM and the CaMBD region of nNOS

**DOI:** 10.3389/fnmol.2024.1461272

**Published:** 2024-09-03

**Authors:** Wei Wan, Nan Wang

**Affiliations:** ^1^Research Center for Biochemistry and Molecular Biology, Jiangsu Key Laboratory of Brain Disease Bioinformation, Xuzhou Medical University, Xuzhou, Jiangsu, China; ^2^National Demonstration Center of Experimental Basic Medical Science Education, Xuzhou Medical University, Xuzhou, Jiangsu, China

**Keywords:** nNOS, CaM, CaMBD, interference peptides, molecular dynamics

## Abstract

**Introduction:**

The neuronal nitric oxide synthase (nNOS) subtype of nitric oxide synthase (NOS) is an enzyme required for learning and memory. Overactivation of nNOS can lead to oxidative/nitrite stress, which is complicit in the pathophysiology of various neurological and psychiatric disorders. Previous studies have shown that calmodulin (CaM) forms complexes with Ca^2+^ and binds to the calmodulin-binding domain (CaMBD) of nNOS, thereby upregulating its catalytic activity in hippocampal neurons. To date, there has been no explanation for the non-covalent interactions in the CaMBD-CaM binding structure model of nNOS.

**Methods:**

In this study, we aimed to investigate the intrinsic factors involved in the binding of CaM to NOS-CaMBD and designed interfering peptides based on the N0 peptide structure of the original nNOS-CaMBD sequence: N1 (obtained from the L734F mutation), N2 (obtained from the F731Y and F740Y mutations), and N3 (obtained from the F731L, V738L, and F740L mutations). We employed homology modeling to construct six CaM-peptide complex models, aiming to elucidate the roles of key amino acid residues within the N0 peptide in its interaction with CaM by means of molecular dynamics simulations. The effect of the peptides on the activation and release of NO by nNOS in neurons was assessed using murine primary neuronal cells.

**Results:**

When measuring neuronal NO content, it was found that adding N2 and N3 to cultivated neurons significantly increased nNOS activity, leading to the increased NO production. We found that interfering peptides could stably bind to CaM. Among them, N2 and CaM exhibited the strongest binding ability, indicating that the polarized benzene ring significantly enhanced the binding between nNOS-CaMBD and CaM. Conversely, the binding ability between N0 and CaM was the weakest, as they exhibited the worst polar contact, weakest hydrogen bonding, and the lowest binding free energy. The simulation results also highlighted several important amino acid residues: The K76 of CaM plays an important role in polar contact and hydrogen bonding formation, the L734 residue suppressed model flexibility to a certain extent and had an adverse effect on the overall binding free energy of the model. These results, compared with the results of cellular NO content, a preliminary verification of the antagonistic competitive mechanism between CaM allosteric activation of nNOS and SUMOylation hyperactivation was performed.

**Discussion:**

In summary, this study explored the ability and mode of action of key residues in nNOS-CaMBD on the binding of interfering peptides to CaM, thereby providing new structural perspectives for the activation of nNOS by CaM and recommendations for drug design targeting the specific inhibition of nNOS.

## Introduction

Nitric oxide synthase (NOS) functions exclusively in its dimeric configuration within the biological milieu, orchestrating the synthesis of its active product, nitric oxide (NO), by converting L-arginine (L-Arg) in the presence of cofactors such as NADPH and tetrahydrobiopterin (BH4) (Palmer et al., 1988). Structurally, the enzyme comprises a C-terminal flavoprotein-rich reductase domain, which is responsible for binding NADPH, FAD, and FMN; a central calmodulin (CaM)-binding domain; and an N-terminal oxygenase domain housing the heme and redox cofactor, (R)-5,6,7,8-tetrahydrobiopterin (BH4)-binding sites, and the substrate-binding site for L-Arg (Li and Poulos, 2005). NOS has three distinct subtypes: neuronal nitric oxide synthase (nNOS), inducible nitric oxide synthase (iNOS), and endothelial nitric oxide synthase (eNOS), all of which exhibit notable sequence homology and are differentially regulated based on cellular localization (Alderton et al., 2001; Li and Poulos, 2005). Despite their divergent regulatory mechanisms, all NOS subtypes possess calmodulin-binding domains (CaMBDs) capable of interacting with CaM, thereby activating NOS and eliciting context-specific functions (Bredt and Snyder, 1990; Abu-Soud et al., 1994; Panda et al., 2001).

Among the triad of NOS subtypes, nNOS is the principal source of NO within neuronal contexts. nNOS is a constitutive enzyme that is prominently expressed in the mammalian brain and skeletal muscles and facilitates the conversion of L-Arg into NO. Existing research underscores the basal activity of nNOS under physiological conditions, which is intricately linked to fundamental biological processes such as cognition, memory, and skeletal muscle function (Kelley et al., 2009; Kelley et al., 2011; Ito et al., 2013; Pavesi et al., 2013; Baldelli et al., 2014; James et al., 2015). Nonetheless, the hyperactivation of nNOS within neuronal environments can precipitate oxidative stress and free radical accumulation, culminating in neurotoxicity implicated in the neuronal death observed in neurological and psychiatric disorders, including hypoxic–ischemic encephalopathy, ischemic stroke, and traumatic brain injury (Yu et al., 2008; Zhou et al., 2010; Yin et al., 2013; Favié et al., 2018; Qu et al., 2020; Wang et al., 2022). Thus, uncovering the structural underpinnings of nNOS activation has profound implications for elucidating its physiological and pathophysiological roles. Each monomeric unit of nNOS predominantly comprises a PDZ domain, oxygenase domain (heme domain), cross-linked helical domain (CaMBD), and reductase domain (Zhou and Zhu, 2009). The CaMBD, situated within the α-spiral region spanning nNOS725-745, represents a basic amphiphilic molecule adept at accommodating CaM binding (Bredt and Snyder, 1990; Bredt et al., 1991), an integral component of calcium signaling pathways that regulate various enzymes, ion channels, aquaporins, and other proteins via calcium-mediated interactions. Structurally, CaM is characterized by four distinct EF-hand domains (EF-hand1–4), each coordinating with Ca^2+^ ions 1–4, respectively (Kumar et al., 2013; Kursula, 2014). The binding between CaM and nNOS-CaMBD facilitates electron flow from NADPH to the flavin reductase domain and, subsequently, to the heme center (Guan and Iyanagi, 2003; Roman and Masters, 2006; Zhou and Zhu, 2009). Notably, research has posited that under low intracellular Ca^2+^ concentrations, CaM dissociates from nNOS, resulting in its inactivation (Li and Poulos, 2005; Costa et al., 2016). Hence, elucidating the structural dynamics of the interplay between CaMBD and CaM during aberrant elevations in Ca^2+^ concentrations holds promise for mitigating the associated brain injuries. Ng HL et al. initially proposed and constructed the X-ray structure (PDB code: 2O60) for nNOS-CaMBD/CaM. Their approach entailed amalgamating peptide fragments spanning positions 725–747 of nNOS with CaM to develop the model, and subsequently investigating the foundational aspects of its behavior. However, a comprehensive exploration of the conformational changes and non-bonding interactions [Polar contact (Zhou and Pang, 2018), hydrogen bonding (Fersht, 1987), and other interactions that contribute to binding free energy (Siebenmorgen and Zacharias, 2019)] accompanying CaM-nNOS-CaMBD complex formation remains elusive and the roles of specific amino acids within nNOS-CaMBD during this process remain unexplored.

Therefore, to further explore the binding dynamics between NOS-CaMBD and CaM and determine the impact of pivotal amino acid residues within nNOS-CaMBD on its affinity for CaM, we initially constructed docking models between NOS-CaMBD and CaM molecules. Furthermore, the nuances of the interaction between CaMBD and CaM were subsequently evaluated using molecular dynamics simulations. Findings from the molecular dynamics simulations underscored that the nNOS-CaMBD residues F731, L734, and F740 played a distinct role in its binding to CaM. Comparative analysis revealed that the binding of interfering peptides N1-N3 to CaM was more stable than that of N0. Previous research conducted in our laboratory identified an alternative form of SUMOylation-mediated covalent activation of nNOS, wherein SUMOylation occurs at the K725 and K739 sites within the CaMBD of nNOS (Du et al., 2020; Wang and Hou, 2020). Based on these conclusions, it can be inferred that a competitive relationship may exist between the two activation modes. Preliminary exploration of the competitive mechanisms was performed by comparing the results of cellular NO content analyses. Future investigations may involve targeted amino acid mutations and exploring the use of specific nNOS inhibitors at the nNOS-CaMBD site. Such endeavors hold promise for designing pharmacological interventions to ameliorate nNOS overactivation, with potential therapeutic implications for managing neurological and psychiatric disorders.

## Results

### The surface of CaM provides a prospective docking site for interactions with interfering peptides

**Figure 1:**
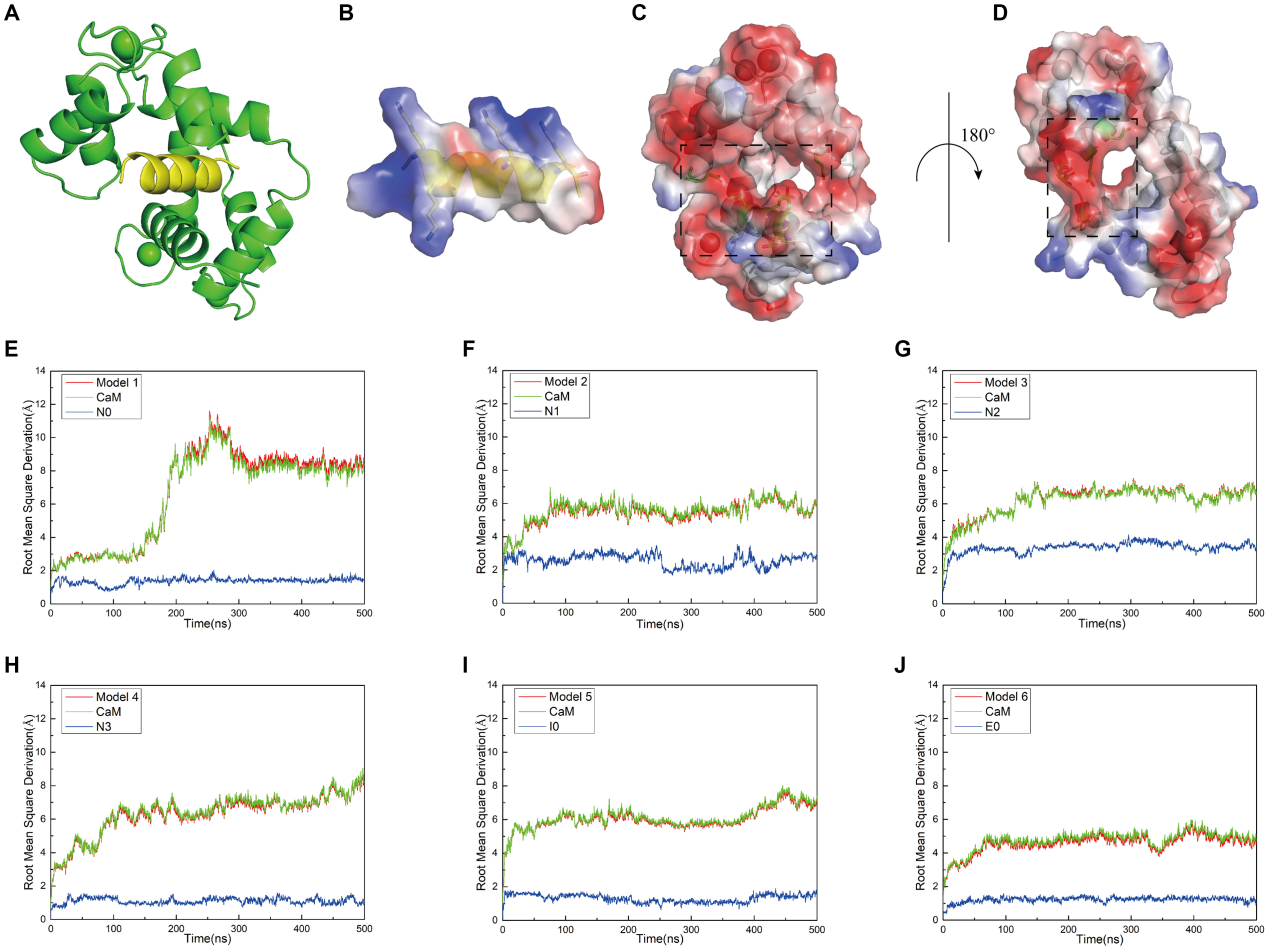
**(A)** Structural model of the docking between interfering peptides (represented by N0) and calmodulin (CaM) molecules. **(B)** A transparent vacuum electrostatic surface illustrating the vacuum electrostatic calculation of the secondary structure of the interfering peptide (N0). Red, white, and blue denote negatively charged, neutral, and positively charged surfaces, respectively. **(C)** A transparent vacuum electrostatic surface depicting the vacuum electrostatic calculation of the secondary structure of CaM. The dashed box indicates the positively charged head of the interfering peptide that facilitates attraction. **(D)** Horizontal rotation of **(C)** by 180° to display the vacuum electrostatic calculation on the back of CaM. The dashed box signifies the positively charged tail of the interfering peptide, promoting attraction. **(E–J)** The relationship between the Root Mean Square Deviation (RMSD) curves of Models 1–6 and simulation time. Simultaneously, the RMSD values for both the receptor and ligand were extracted throughout the entire simulation process to generate RMSD curves. The red, green, and blue curves represent the RMSD curves of the global structure, interfering peptide, and CaM post-docking with the interfering peptide, respectively.

To investigate whether the peptide sequence in CaMBD can mitigate nerve damage by modulating the interaction between CaM and nNOS-CaMBD, our laboratory conducted experiments using the original N0 sequence to assess its impact on cellular NO content. The results indicated that N0 had minimal effect, leading us to preliminarily speculate that this might be due to insufficient interaction between the molecules. To further explore this, vacuum electrostatic surface calculations were performed on N0. Analysis of the charge distribution on the surface of N0 (Figure 1B) revealed significant non-bonding interactions, particularly hydrophobic interactions. Notably, the free energy allocation results for N0 indicated that residues F731 and V738 contributed minimally to van der Waals interactions, whereas L734 and F740 made substantial contributions to the binding energy of N0. Based on these findings, we conducted mutation studies to observe changes in the binding affinity of interfering peptides to CaM. As a reference, we utilized the crystal structures of nNOS-CaMBD/CaM, iNOS-CaMBD/CaM, and eNOS-CaMBD/CaM, obtained from the RCSB database, for further modeling. By employing homologous modeling, we docked the rat nNOS-CaMBD 731–744 peptide (N0), iNOS-CaMBD 515–526 peptide (I0), and eNOS-CaMBD 494–507 peptide (E0) with CaM to generate models 1 (N0/CaM) (Figure 1A), 5 (I0/CaM), and 6 (E0/CaM), respectively. Subsequently, we introduced mutations to several key amino acids within N0, resulting in the creation of three novel interfering peptides, designated as N1, N2, and N3. Specifically, the fourth amino acid L in N0 was subjected to a hydrophobic mutation to F, yielding N1; the polarized side chain benzene ring of the first amino acid F was mutated to Y, alongside a similar mutation for the tenth amino acid F, resulting in N2. In the following discussion, we refer to the hydroxyl-substituted benzene ring (R group of tyrosine) as a “polarized benzene ring,” given that the polarity of this substituted benzene ring is greater than that of a regular benzene ring. The first amino acid F, eighth amino acid V, and tenth amino acid F were replaced with L to yield N3. We conducted computational simulations and compared the results with the N0 peptide, observing slight conformational differences among the interfering peptides, though their overall structures remained similar (Supplementary Figure S1). To ensure peptide solubility and maintain consistency with cellular experiments, we ultimately docked the uncapped peptides with CaM to construct the final model. Three additional interference peptide/CaM docking models were established: Model 2 (N1/CaM), Model 3 (N2/CaM), and Model 4 (N3/CaM).

When investigating the rationale for CaM binding to NOS-CaMBD, we observed that the vicinity surrounding N0, encompassing amino acid residues K2, K3, K9, and K13 (corresponding to K732, K733, K739, and K743 in nNOS-CaMBD, respectively), contained a substantial number of positive charges. Similar surface charge distributions were observed for other interfering peptides. In contrast, CaM adopted a concave structure characterized by a negatively charged surface at its head (Figure 1C), attributed to amino acid residues E7, E8, E12, E15, E85, and E115, and a corresponding negatively charged surface at its tail (Figure 1D), comprising amino acid residues D79, D81, E85, and E88. Owing to the divergent charge distributions on these two surfaces, N0 seamlessly docked within the crevices of CaM, establishing mutual attraction through intermolecular electrostatic interactions. This established the foundation behind non-bonding interactions between NOS-CaMBD and CaM. However, the presence of residue E6 in N0 (corresponding to E736 in nNOS-CaMBD) also contributed to the generation of negatively charged surfaces, thereby exerting repulsive forces against the concave structure of CaM. This phenomenon may provide a partial basis for the conformational adjustments observed during the binding of CaM to nNOS-CaMBD. These docking results, complemented by vacuum electrostatic surface calculations, provide a substantive basis for exploring the activation mechanism of NOS by CaM via non-bonding and NOS-CaMBD interactions.

### Interfering peptides demonstrate stable binding affinity with CaM

Initially, molecular dynamics simulations were conducted to assess the relative stability of the combined models. Root-mean-square deviation (RMSD) calculations revealed that equilibrium was reached in all six models after approximately 500 ns. Examination of the RMSD diagrams for Models 1–6 (Figures 1E–J) indicated stable binding of the interfering peptides to CaM, thereby providing corroborative evidence for the establishment of our models. Notably, Models 1–4 exhibited less fluctuation in the RMSD curves of N0, N2 and N3, suggesting enhanced stability upon their combination. Across all the models, the RMSD values for the interfering peptide segments were generally lower than those for the CaM segments, indicating similar fluctuation patterns and magnitudes in the RMSD curves between the overall model and the CaM portion. Notably, significant fluctuations in the RMSD values of the CaM region suggested conformational changes from the original state throughout the simulation process.

**Figure 2:**
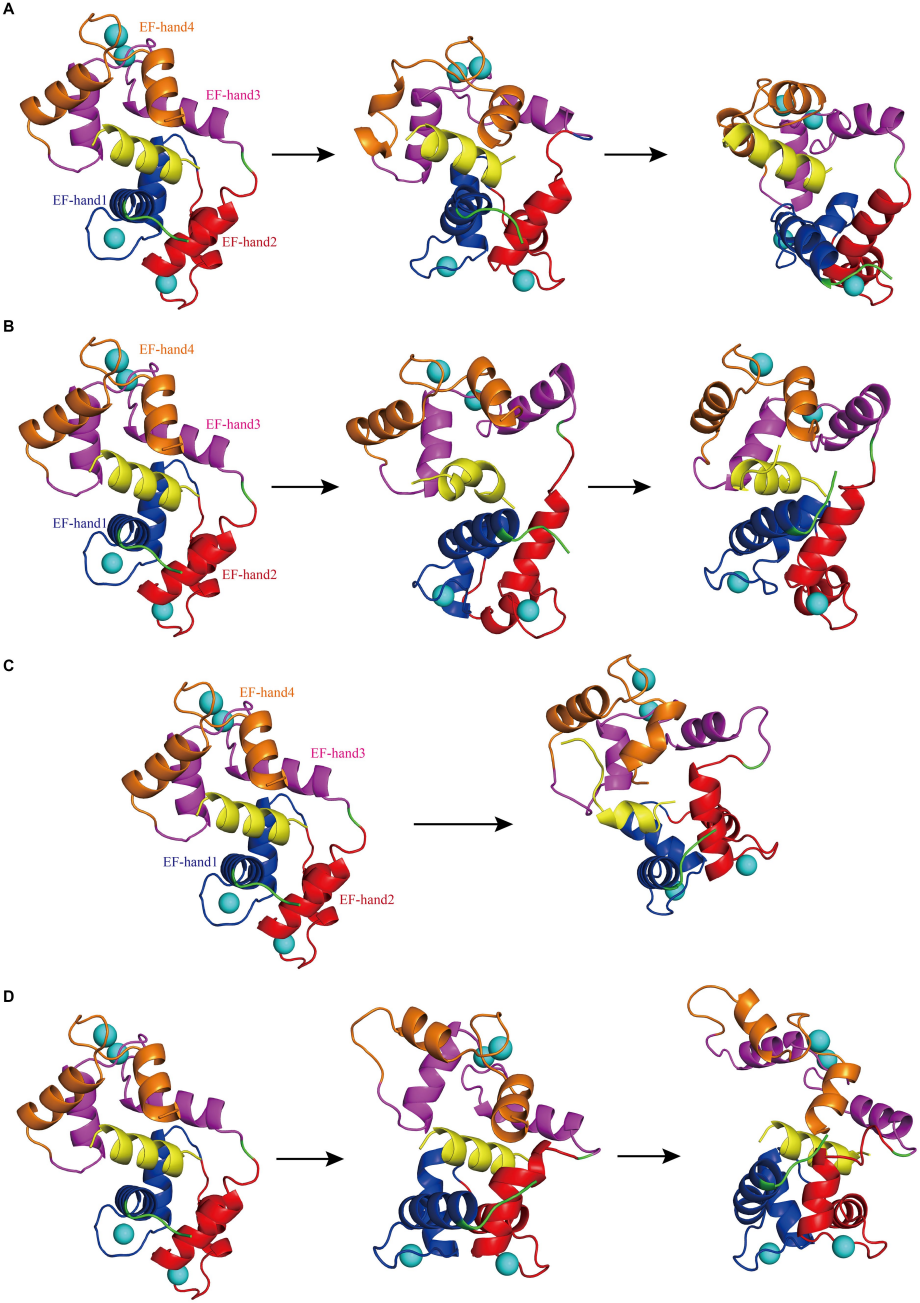
**(A)** The first panel depicts the initial structural diagram of Model 1, where the EF-hand1 (blue region), EF-hand2 (red region), EF-hand3 (pink region), and EF-hand4 (brown region) domains of CaM, interfering peptide (yellow region), and Ca^2+^ (the blue sphere) are shown. The second panel illustrates the secondary structure diagram of Model 1 at 93 ns during the simulation. The third panel shows the secondary structure diagram of Model 1 at 400 ns during the simulation. **(B)** The first panel displays the initial structure diagram of Model 2. The second panel shows the secondary structure diagram of Model 2 at 275 ns during the simulation. The third panel displays the secondary structure diagram of Model 2 at 500 ns during the simulation. **(C)** The first panel presents the initial structure diagram of Model 3. The second panel illustrates the secondary structure diagram of Model 3 at 500 ns during the simulation. **(D)** The first panel presents the initial structure of Model 4. The second panel demonstrates the secondary structure diagram of Model 4 at 350 ns during the simulation. The third panel displays the secondary structure diagram of Model 4 at 500 ns during the simulation.

While the RMSD results for Models 1–4 indicated that equilibrium was reached after 500 ns, noteworthy discrepancies were observed between the final RMSD curves and those obtained at earlier time points. The varying terminal RMSD values among these models suggested that the conformational changes induced by the docking of interfering peptides with different sequences were not uniform, with some models exhibiting more pronounced alterations. The conformational changes in Models 1–4 were further explored. Initially, by scrutinizing the RMSD curves, it was noted that in Model 1, the CaM segments reached equilibrium after 25 ns, ultimately attaining a stable conformation by 93 ns (Figure 2A). Subsequent analysis revealed significant fluctuations in the RMSD curve of CaM between 150 and 300 ns, stabilizing at 7–9 Å, indicating further conformational change and the attainment of a new equilibrium point. Model 1 ultimately attained a new conformation within 400 ns (Figure 2A), with discernible conformational changes observed in the EF-hand helix compared to the initial state, subsequently gradually releasing N0. In Model 2, the RMSD curve rose sharply from 0 to 77 ns, stabilizing at 5–7 Å, similar to the relatively stable conformation observed at 275 ns (Figure 2B). The RMSD curve of the interfering peptide region N1 oscillates frequently, indicating frequent conformational changes and ultimately reaching equilibrium at the end of the simulation, similar to the relatively stable conformation observed at 500 ns (Figure 2B). Notable conformational changes were observed in EF-hand3/4, indicating its gradual separation from EF-hand1/2 and the subsequent release of N1. In Models 3, the RMSD curves exhibited a fluctuate between 0 and 165 ns and subsequently stabilized at 6–8 Å, similar to the relatively stable conformation observed at 500 ns (Figure 2C), with terminal RMSD values of 6.7 Å. Similarly, in Models 4, the RMSD curves exhibited a sharp fluctuate between 0 and 160 ns and subsequently stabilized at 6–8 Å, similar to the relatively stable conformation observed at 350 ns (Figure 2D). However, CaM exhibited slight oscillations toward the end of the simulation, ultimately stabilizing at a terminal RMSD value of 8.2 Å, similar to the relatively stable conformation observed at 500 ns (Figure 2D). Remarkably, in Models 3 and 4, EF-hand3/4 exhibited pronounced movement away from EF-hand1/2 (Figures 2C,D), creating an “open mouth” that facilitated the release of interfering peptides, indicative of enhanced stability in the N2/N3 structure. This observation, coupled with the RMSD curve analysis, suggests that the model structure exhibits a more stable conformation.

### Mutations in key amino acids within N0 can potentially enhance NO content in neurons

**Figure 3:**
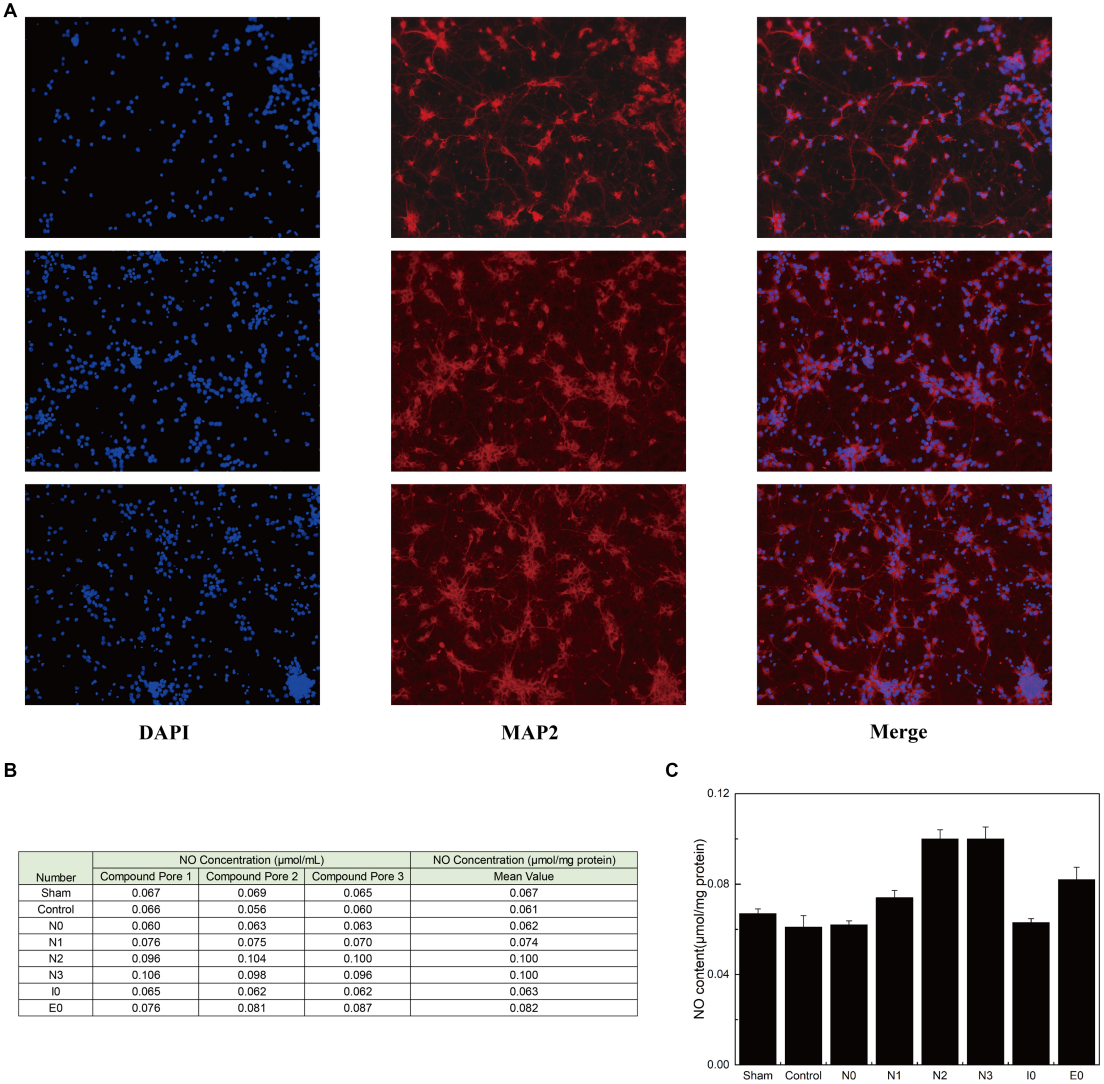
**(A)** Three parallel groups of primary mouse neurons, isolated from mouse brain tissue, were imaged and arranged from top to bottom. Each horizontal row of three images represents the same group of cells stained with DAPI (to label the nuclei, confirming the presence of cells), MAP2 (to label neurons, confirming their neuronal identity), and a merged image showing the combined results of both stains. **(B)** The three groups of cells were evenly divided into eight portions for peptide treatment. Each portion received one of the following treatments: sham surgery (physiological saline), control (peptides with disordered amino acid sequences similar to N0), N0, N1, N2, N3, I0, or E0. The production of NO was quantified using an assay kit. **(C)** The standard deviation of NO content in each group of neurons is listed.

After confirming the stable binding of each interfering peptide to CaM, the effect of the peptides on the activation and release of NO by nNOS in neurons was assessed using murine primary neuronal cells. Initially, immunofluorescence verification was performed on primary neuronal cells to determine their purity (Figure 3A). Subsequently, the cells were incubated using the corresponding interfering peptides, and the NO content in the neurons was measured. Figures 3B,C show that there was no significant difference in NO content when neurons were cultured with control peptides (Control), N0, or I0 compared to that in the baseline physiological saline group (Sham). However, the NO content in the N1 group was slightly higher than that in the sham group, with statistical significance (*p* < 0.05). Moreover, NO content in the E0 group was significantly higher than that in the sham group (*p* < 0.01). Notably, the NO content in the N2 and N3 groups increased the most substantially compared to that in the Sham group, with a significant difference (*p* < 0.001). These findings suggest that the addition of N2 and N3 to cultured neurons significantly enhanced NO content, indicating their ability to augment nNOS activity and promote NO production. To further investigate the underlying factors influencing the binding of CaM to NOS-CaMBD and determine whether differences in cellular NO content were attributable to structural variations in Models 1–4, trajectories were obtained from the equilibrium stage of the RMSD. A post-simulation analysis was conducted to calculate the contribution of non-bonding interactions in the six models.

### Interfering peptides exhibit stable binding to CaM, as verified through polar contact analysis and hydrogen bonding assessments

**Figure 4:**
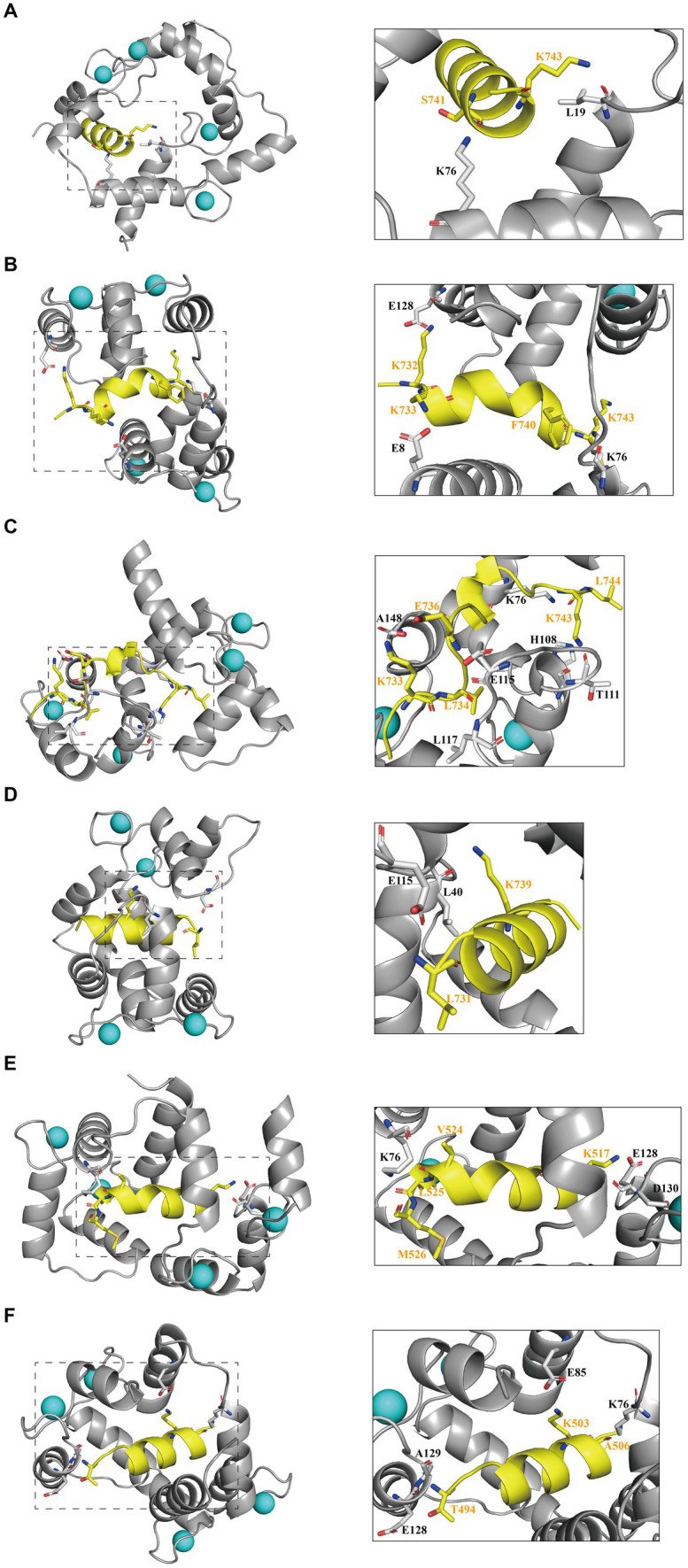
The first panel for **(A)** Models 1, **(B)** 2, **(C)** 3, **(D)** 4, **(E)** 5, and **(F)** 6 displays the secondary structure diagram of the equilibrium end during the simulation process. The second panel illustrates the residues involved in intermolecular polar contacts (interfering peptides/CaM), delineated by dashed boxes for Models 1/2/3/4/5/6. CaM residues are depicted in black, whereas interfering peptide residues are depicted in yellow.

To elucidate the primary interactions that regulate the binding of interfering peptides to CaM in molecular docking and to distinguish differences in RMSD curves and values across the various models, polar contact studies and statistical analyses of hydrogen bonding were performed. Initially, by analyzing molecular dynamics simulations, unique intermolecular polar interactions were observed in the end structures of Models 1–4 following the modification of key amino acids in N0 (Figures 4A–D). In Model 1–3, the K76 residue of CaM was engaged in intermolecular polar contacts, underscoring the significance of K76 residues in the binding of interfering peptides to CaM. In Model 1, other than K76 and L19 of CaM, no other residues exhibited polar contacts with N0. The reduced number of interaction sites in Model 1 led to pronounced fluctuations in the RMSD curve compared with the interfering peptides in Models 2/3/4. In Model 2, CaM observed that E128, E8, K76 established polar contact with the first and last residues of N1, which may be a partial reason for the frequent conformational changes of the middle residues of N1 during the simulation process. In Models 3, numerous polar contacts were established between the interfering peptides and CaM, resulting in diminished fluctuations and improved stability in the RMSD curves of Model 3 following binding. In the CaM of Model 4, only E115 and L40 have established polar contact with N3, but the RMSD curve of Model 4 has a small fluctuation amplitude, suggesting the presence of additional key influencing factors. To further explore the polar interaction between CaM and NOS-CaMBD molecules and validate the aforementioned findings, a polar contact analysis of the end structures of Models 1, 5, and 6 was conducted (Figures 4A,E,F). K76 was identified to be involved in intermolecular polar contact in all three models, confirming its pivotal role as a key amino acid residue in the activation of the NOS structure induced by the binding of CaM to CaMBD. Moreover, the interfering peptides in Models 5 and 6 exhibited increased polar contact with CaM, which explains the enhanced stability observed in their RMSD curves compared to Model 1 following initial conformational changes.

**Figure 5:**
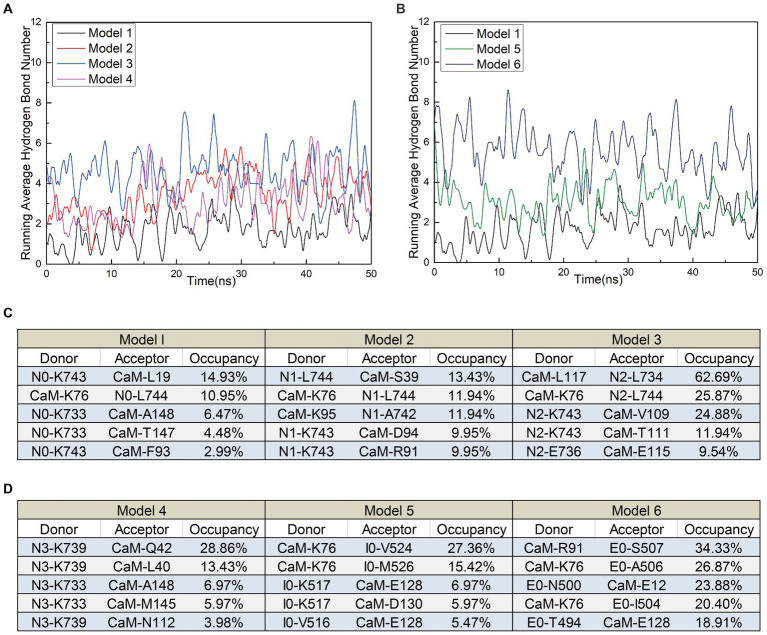
**(A)** The number of hydrogen bonds over time for Models 1, 5, and 6. **(B)** The number of hydrogen bonds over time for Models 1, 2, 3, and 4. **(C,D)** Detailed hydrogen bond occupancy in Models 1–6, including listed donors and recipients.

In addition to polar contacts, hydrogen bonds play a crucial role in protein–protein interactions. To further investigate the intermolecular hydrogen bonding in interactions between interfering peptides and CaM, hydrogen bonding statistics were analyzed, considering a truncation distance of 3.5 Å and a truncation angle of 120°. The total number of hydrogen bonds and the most important hydrogen bonds with the top five highest occupancies are listed in Figures 5A–D, respectively. Throughout the simulation process in Models 1–6, the number of hydrogen bonds in Models 2, 3, 4, 5 and 6 increased by approximately 103.5, 175.3, 84.2, 79.2 and 229.6% respectively, compared to Model 1. In Model 2, the maximum occupancy rate of the first 5 hydrogen bonds is less than 20%. Model 3 detected approximately 3–8 hydrogen bonds throughout the entire simulation process, with small fluctuations in the number of hydrogen bonds. The high number of hydrogen bonds in Model 4 helps to stabilize its conformation to a certain extent. Model 6 has the highest number of hydrogen bonds and the most important hydrogen bond occupancy rate is 18–34%. Therefore, the RMSD curve of Model 6 is the most stable and the equilibrium conformation is very stable. Notably, while the geometric configuration suggests the potential existence of these hydrogen bonds, their proportion in the N0/N1 complexes is very low. However, to enable a comprehensive comparison between N0 and the other peptides, we included an analysis of these hydrogen bonds as well. Meanwhile, the CaM residues K76 were found to play important roles in hydrogen bond formation within the models. Except for Model 4, the five most significant hydrogen bonds with the highest occupancy rates in each model were formed by these residues (Figures 5C,D).

These findings suggest that stable polar contacts and hydrogen bonds between CaM and NOS-CaMBD enable stable binding, with residues K76 of CaM playing crucial roles. Models 1–6 exhibited varying degrees of stability, polar contact, and hydrogen bonding. This highlights the importance of comprehensive data analysis that takes into account contributions from both the main-chain and neutral residues to reveal differences in binding effects with CaM caused by interfering peptide amino acid residues across the four models.

### The mutation of nNOS residue L734F enhances the flexibility of model 2

**Figure 6:**
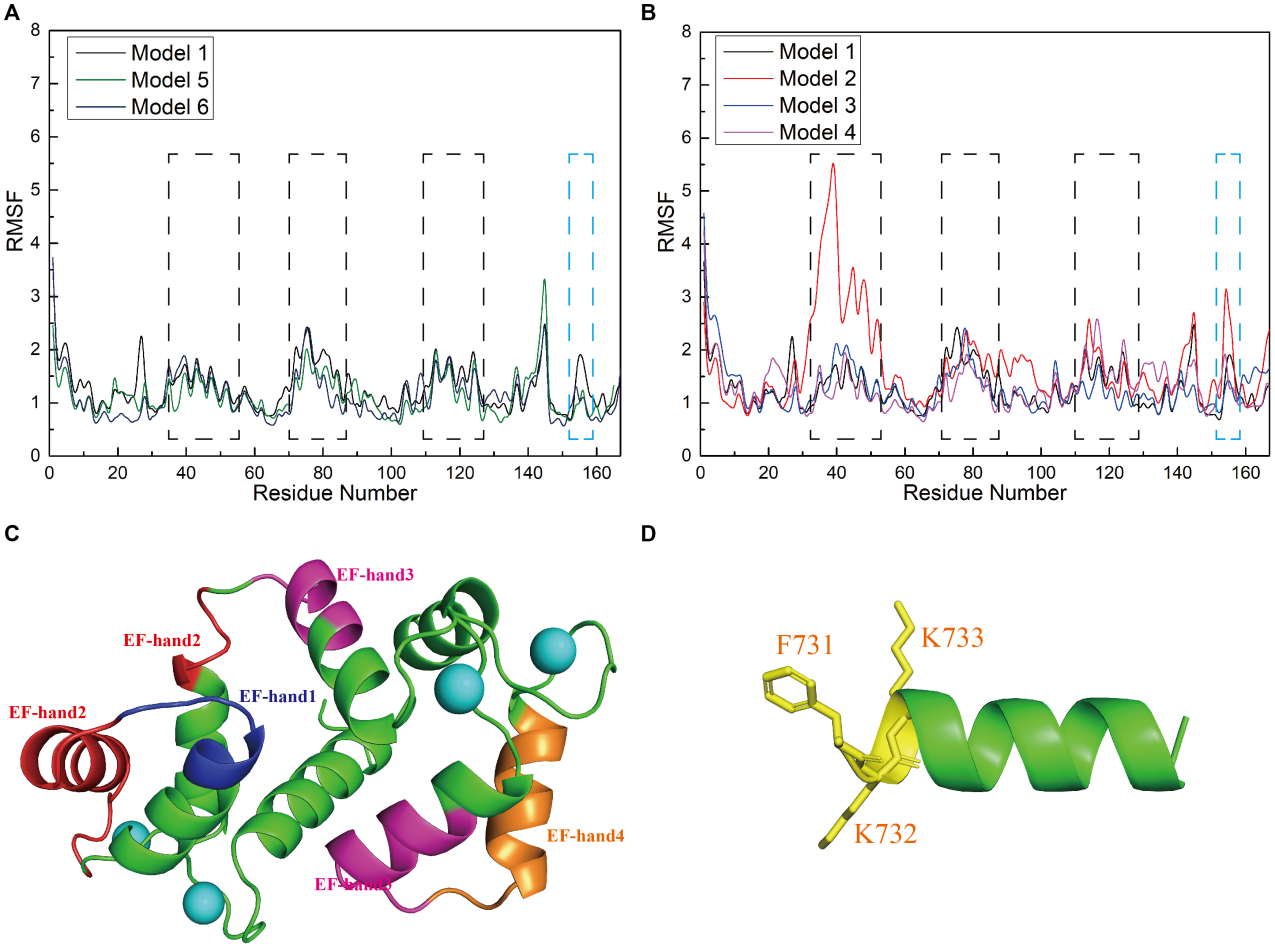
**(A,B)** Root mean square fluctuation (RMSF; Å) calculations of interfering peptide/CaM residues in each model. The RMSF values (Å) of Models 1–6 are depicted using black/red/blue/magenta/yellow-brown/navy blue curves, respectively. **(C)** Structural depiction of black planar residues in CaM. The blue/red/pink/brown planar structures correspond to the planar residues with black dashed lines in **(A,B)**, situated in the EF-hand1/2/3/4 domains of CaM, respectively. **(D)** Structural depiction of blue planar residues in the interfering peptides (using N0 as an example). The yellow planar structure represents the planar residues depicted with blue dashed lines in **(A,B)**.

To investigate how the amino acid sequences of the interfering peptides affect the flexibility of CaM amino acids, we calculated the root mean square fluctuation (RMSF) values of Models 1–6 and performed a conformational analysis. This analysis aimed to investigate the global structural characteristics of the interaction between interfering peptides and CaM in the six models and to demonstrate how interfering peptides regulate the global structure of CaM. The RMSF maps of Models 1, 5, and 6 revealed that the main flexible areas of the CaM overlapped substantially, with no significant differences in RMSF values. However, in the main flexible areas of the interfering peptides, the RMSF values of I0 and E0 were lower than those of N0 (Figure 6A). Further exploration of flexibility changes was conducted by observing the RMSF curves of Models 1–4 (Figure 6B). In Model 1, amino acid residues with higher RMSF values in the CaM region were primarily located in specific regions. Similar observations were made for Models 2, 3, and 4, in which the flexible regions of CaM were identified primarily within the EF-hand domains (Figure 6C). Notably, Model 1 exhibited fewer polar contacts/hydrogen bonds but had a lower RMSF value in its CaM region, suggesting that additional factors stabilize its structure. Furthermore, Model 2 showed a significant increase in RMSD in the flexible region of EF hand1/2, indicating that the L734F mutation increased flexibility in this region and resulted in more conformations. The main flexible regions of the interfering peptides in Models 1–4 were consistently identified (Figure 6D), with N1 exhibiting higher RMSF values than those for N0, N2 and N3. This observation aligns with the larger oscillations in the RMSD curves observed for N1. These findings further support the validity of the proposed model.

### Correlation factor analysis of global residue interactions in the interfering peptide/CaM complex

**Figure 7:**
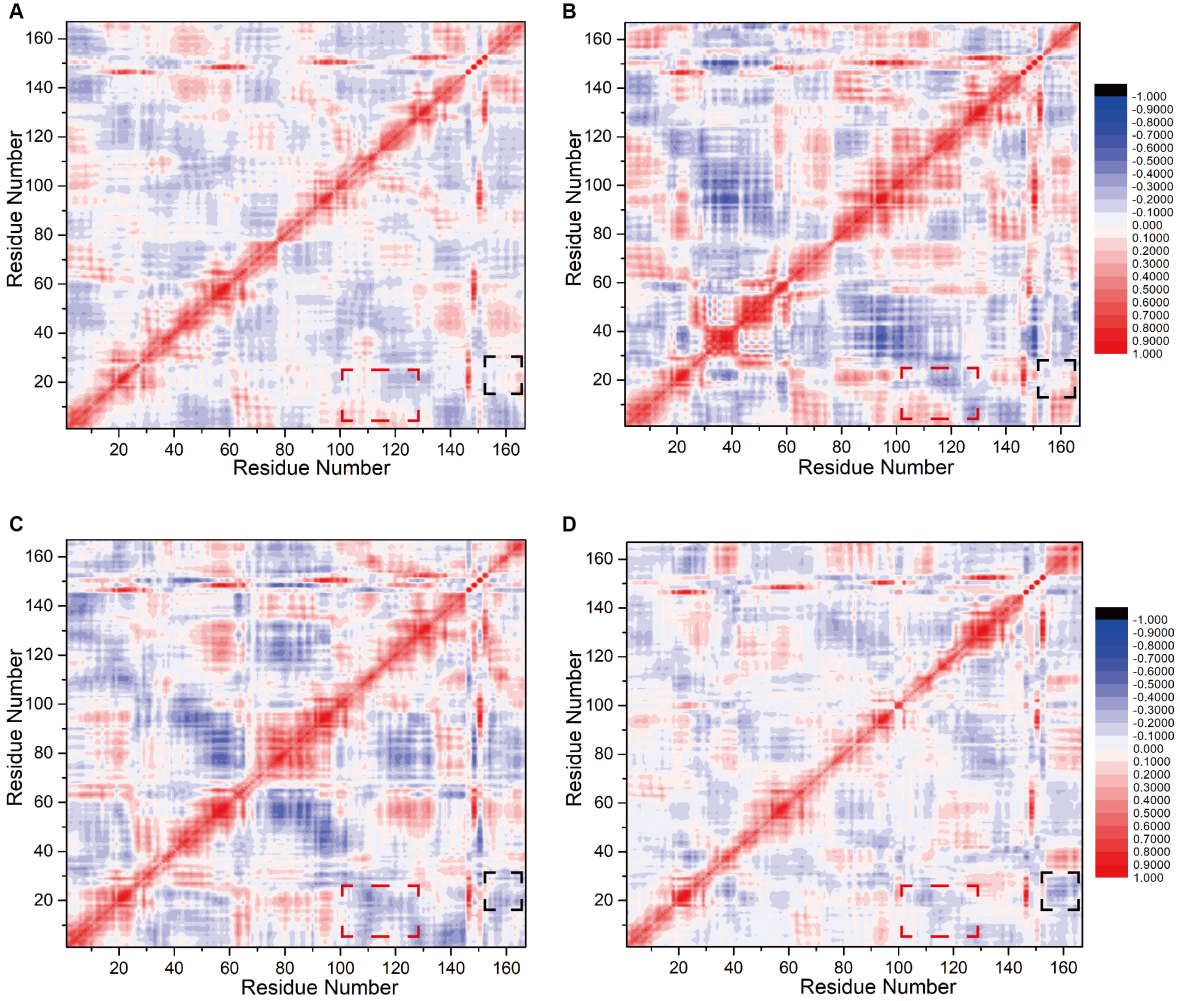
Calculation of correlation factors for **(A)** Models 1, **(B)** 2, **(C)** 3, and **(D)** 4. Highly correlated/anticorrelated residues are indicated in red/blue, respectively. The legend is displayed to the right of **(B)**/**(D)**. The black and red boxes represent the interfering peptide/CaM-related factors discussed in the main text.

To assess the influence of N0 sequence alterations on the internal correlation between the interfering peptides and CaM, we computed inter-residue correlation coefficients reflecting the vibrational characteristics of each amino acid residue in the interfering peptides and CaM. This analysis aimed to elucidate the relationship between amino acid fragments and populations across Models 1–4. Initially, it was noted that owing to the binding of CaM and interfering peptides across all four models and the fact that only select amino acids differed between the interfering peptides, their correlation coefficient graphs (depicted in Figures 7A–D) exhibited similarities, albeit with slight variations in the distribution of correlation factors. Specifically, the sections highlighted with red boxes (comprising residues 2–17 and 100–120, corresponding to amino acid sequences 5–20 and 103–123 in CaM, respectively) in Models 1 and 2 demonstrated positive values. This suggests that these two residue groups may exhibit positive correlation change. In contrast, in Models 3 and 4, the sections highlighted with red boxes exhibited a notable negative correlation, indicating that, under the influence of N2 and N3, there may be a conformational change in the distance between EF-hand1 and EF-hand3/4 in CaM. This is consistent with the conformational results of the model.

The sections highlighted in black boxes in Model 1and 2 (comprising residues 15–25 and 154–167, corresponding to amino acid sequences 18–28 in CaM and 731–744 in nNOS-CaMBD, respectively) also exhibited near-neutral correlations with slightly positive values. This finding suggests that these two residue groups may not have undergone significant global or positive correlation changes. Conversely, in Models 3 and 4, the portions highlighted with black boxes exhibited a significant negative correlation, with the two amino acid residue groups moving closer together. This indicates that mutations in the key amino acid residues of N0 lead to changes in the overall conformation. N2 and N3 may be far from the head end of EF hand1 in CaM, keeping the interfering peptide in the middle of the cavity, stabilizing non bonding interactions with various parts of CaM, and maintaining a more stable equilibrium conformation. Compared to Model 1 and 2, mutations in the interfering peptides in Models 3, and 4 appeared to be more advantageous. In summary, balance conformational analysis based on the model, the correlation factor analysis between amino acid fragments and populations in the four models indicated that the binding effect of CaM and N0 in Model 1 was suboptimal, which was consistent with the findings of previous polar contact and hydrogen bonding analyses.

### Free energy calculation provides valuable insights into the interfering peptide/CaM interaction

**Figure 8:**
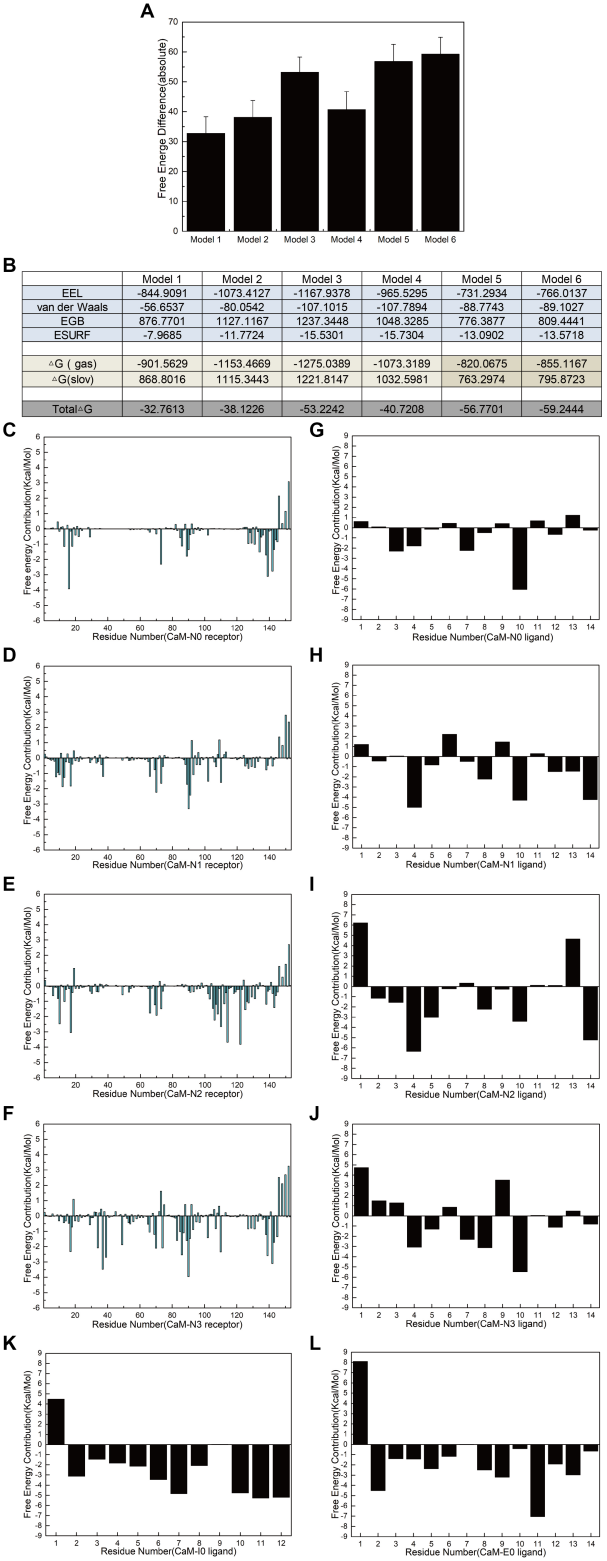
Calculation and decomposition of the free energy of each model. **(A)** The standard error for the absolute bound free energy of Models 1–6 is listed. **(B)** The components of free energy in the calculation are listed. **(C–F)** Energy decomposition diagrams of CaM in the receptor region of Models 1–6. **(G–L)** Energy breakdown diagrams of the ligand interference peptides in Models 1–6. All units are in kilocalories per mole.

Polar contact analysis, hydrogen bonding statistics, and correlation coefficient analysis offer a foundational understanding of how individual amino acid residues or groups contribute to interfering peptide/CaM interactions. However, certain pivotal amino acid residues that influence peptide/CaM interactions remain challenging to elucidate, primarily because analytical approaches only partially consider the characteristics of the amino acid residues. Moreover, essential interactions such as van der Waals interactions necessitate additional investigation, which can be achieved through the application of free energy calculations. To address this gap, we performed binding free energy calculations on the six models in implicit solvents using interfering peptides as ligands and CaM as a receptor. Notably, Models 5 and 6 exhibited significantly higher binding free energies than Model 1, indicating that combining CaM with NOS-CaMBD was advantageous in terms of energy (Figure 8A). Analysis of the combined energy of Models 1–4 revealed varying degrees of decrease, with Model 1 exhibiting the smallest decrease, whereas Models 2, 3, and 4 exhibited larger decreases than Model 1. Specifically, Model 3 exhibited the largest decrease, indicating a heightened energy advantage (Figure 8A). Furthermore, compared to Model 1, Models 2 and 3 demonstrated enhanced electrostatic interactions (EEL) and contributed more binding free energy, consistent with the findings of the polar contact analysis. In addition, in Model 3 and 4, the van der Waals interactions played a more significant role, underscoring their importance (Figure 8B), which is closely related to the hydrophobicity mutation we conducted.

Additionally, to further elucidate the key residues involved in peptide/CaM interactions across Models 1–4, the binding free energy results were analyzed, with the contribution of each residue examined (Figures 8C–J). The findings revealed notable contributions from specific residues in each model. In Model 2, residues M73, F93 and D94 of the CaM protein receptor; in Model 3, residues F20, L117, and M125 of the receptor; and in Model 4, residues L40, N42, and F93 of the receptor each substantially contributed over 2 kcal/mol of binding free energy in their respective models. Intriguingly, the contributions of these residues were either absent or minimal in Model 1. This observation suggests that mutations corresponding to N0 may confer advantageous effects by interfering with the binding of peptides and CaM. The contribution of amino acid residues from the interfering peptide ligands varied across the models. Notably, the residues at positions 4 and 10 emerged as crucial contributors to binding free energy across all four models. Furthermore, when F731 in N2/N3 was mutated, the first residue demonstrated notably unfavorable contributions to the binding free energy. To further determine the impact of F731 on the binding free energy, the free energy contributions of ligand residues I0 and E0 were calculated in Models 5 and 6, revealing adverse effects attributable to their first amino acid residues, L515 and T494, respectively (Figures 8K,L). This underscores the role of F731 in enhancing the binding free energy of the interference peptide, although its overall impact on the free energy of the model requires further validation. These findings provide corroborative evidence for mutations within the CaMBD precursor sequence N0 of nNOS.

Comparative analysis of the ligand-binding free energies between Models 1 and 2 revealed that the hydrophobic mutation L734F significantly increased the overall binding free energy contribution of the ligand by 46.3%, indicating that L734 had an inhibitory effect on the binding energy of interfering peptides and the overall free energy of Model 1. Similarly, a comparison between Models 1 and 3 revealed that the polarization of the benzene ring on the side chains of F731 and F740 only augmented the overall binding free energy contribution of the ligands by 14.7%. But the van der Waals interaction of N2 significantly increases the contribution of bound free energy to Model 3 by 89%. Finally, a comparison of the binding free energies of the ligands in Models 1 and 4 indicated that the hydrophobicity mutations F731L, V738L, and F740L reduced the overall binding free energy contribution of the ligands by 53.7%. However, its van der Waals interaction contributes 90.2% more to the bound free energy of Model 4. This is also consistent with the 3/4 mutation idea of the model and the overall bound free energy results of the model. These findings highlight the critical roles of the residues involved in polar contacts/hydrogen bonds and those that facilitate van der Waals interactions in interfering with peptide/CaM interactions.

## Discussion

In this study, we constructed six CaM-peptide complex models using homology modeling to examine the binding interactions between interference peptides and CaM through a range of analytical techniques, including RMSD, polar contact analysis, hydrogen bonding statistics, RMSF, correlation coefficient analysis, and free energy analysis. Additionally, we assessed intracellular NO content following neuronal culture with interference peptides. These analyses provide insight on the intrinsic factors governing the binding of CaM within the CaMBD, even in scenarios where the amino acid sequences of CaMBD in nNOS are not entirely identical. Our findings highlight the significance of specific amino acid residues within the CaMBD of nNOS in mediating its binding with CaM. This differential interaction leads to distinct conformational changes in CaM when influenced by corresponding interfering peptides, subsequently affecting the activation and release of NO by nNOS. These insights offer valuable implications for investigating the mechanisms of CaM activation in other proteins. Furthermore, considering the competitive mechanism between the allosteric activation of nNOS by CaM and SUMOylation-mediated overactivation of nNOS in the CaMBD region, our results provide valuable insights for the further design and refinement of specific nNOS inhibitors targeting SUMOylation. Such efforts are promising for uncovering novel therapeutic strategies aimed at addressing neurological and psychiatric disorders stemming from excessive nNOS activation.

The precise mechanism underlying the activation of NOS by CaM remains unclear, with research indicating that this interaction is modulated by various post-translational modifications, including phosphorylation (Bredt et al., 1992; Piazza et al., 2015). In addition, non-covalent interactions play a significant role in inducing global conformational changes. Using molecular dynamics simulations, we demonstrated that the CaMBD of NOS forms complexes with CaM primarily through non-bonding interactions. Crystallographic models depicting the binding of CaM to NOS-CaMBD in databases provide a foundational framework for investigating the intrinsic determinants of CaM binding within the CaMBD region and aid in elucidating the influence of key neuronal nNOS-CaMBD amino acid residues on its binding affinity.

Simulation calculations encompassing polar contacts, hydrogen bonding, and other non-bonding interactions that contribute to the binding free energy in Models 1, 5, and 6 demonstrated that CaM provides an electrostatic docking region that forms stable associations with the CaMBD region of NOS via polar contacts, hydrogen bonding, and binding energy. This empirical evidence supports the current understanding of the specific binding of CaM within the CaMBD region of NOS and highlights the significance of non-bonding interactions, particularly electrostatic interactions, which typically facilitate remote interactions (Shashikala et al., 2019). Such interactions tend to occur earlier or have greater strength than other protein-binary interaction structures, such as NOS-CaMBD/CaM (Yang et al., 2020). In the investigation, it was observed that the binding capability of Models 2, 3, and 4 significantly surpassed that of the original sequence N0 within the CaMBD of nNOS. Notably, in Model 3, the N2/CaM binding exhibited superior polar contacts, van der Waals interactions, and binding free energy, consequently forming the most stable interaction with the strongest binding affinity. This finding aligns with our cell experiment results, which demonstrated that due to the weak binding affinity between N0 and CaM, there was no significant difference observed between the N0-treated group and the Sham or Control groups. This study highlights that the polarization of the benzene rings of residues F731 and F740 within N0 substantially enhances its binding capacity with CaM. Furthermore, key residues involved in the interaction between nNOS-CaMBD and CaM were identified. Specifically, L734 in nNOS-CaMBD has a certain inhibitory effect on the flexibility of the model and has an adverse effect on the overall binding free energy of the model. In addition, there are some amino acids worth exploring in subsequent experiments, such as K76 residue in CaM.

In cellular experiments, we observed that the results of the Sham group, Control group, and N0 group were similar. Computational simulations further support the conclusion that the binding affinity between N0 and CaM is undetectable, which corresponds with the cell experiment findings. In contrast, Model 3, Model 4, and Model 6 significantly increased NO content in mouse neurons. We tentatively attribute this phenomenon to the competitive interactions between interfering peptides and CaM for binding. However, it is important to consider the antagonistic relationship between SUMOylation-mediated overactivation and allosteric activation by CaM, as both compete to bind to the neuronal nNOS-CaMBD region (Wang and Hou, 2020). The binding of CaM to this region may mitigate excessive SUMOylation of nNOS, potentially leading to decreased NO release. Preliminary confirmation of this hypothesis was obtained from molecular simulations, particularly in Model 3, in which N2 exhibited the strongest binding affinity for CaM. Notably, the altered binding free energy observed in Model 4 compared to Model 2 could be attributed to the hydrophobic mutation of N3. However, it is crucial to acknowledge that this mutation may not fully reflect the influence of entropy on binding energy and may enhance CaM binding through hydrophobic interactions. Interestingly, despite the E0 amino acid sequence in Model 6 being distinct from that of N0, it demonstrated favorable polar contacts, hydrogen bonding, and binding energies, leading to the observed findings. Conversely, although I0 in Model 5 exhibited satisfactory polar contacts and binding energies, it did not increase NO release from the neurons. This discrepancy may stem from the irreversible binding of iNOS to CaM in a Ca^2+^-independent manner, indicating that the binding of CaM to the CaMBD region is influenced by additional factors (Abu-Soud et al., 1994; Förstermann et al., 1994; Panda et al., 2001). These findings support the further exploration of the two competitive activation mechanisms of nNOS to validate these hypotheses and offer valuable insights for future research. In summary, we preliminarily concluded that the polarization mutation of the benzene ring residues F731 and F740 within the amphiphilic spiral CaMBD of nNOS to tyrosine (Y) can significantly enhance their binding affinity to CaM, primarily through polar contacts and binding energy. Additionally, hydrophobic mutations in the corresponding amino acids of nNOS-CaMBD may augment its binding affinity for CaM by increasing the polar contacts and hydrophobic interactions.

Theoretical analysis of the NO content results and the effect of the CaMBD amino acid sequence in nNOS on its interaction with CaM provides novel insights into potential pathological pathways for inhibiting nNOS in cognitive decline and myopathy (Kelley et al., 2009; Kelley et al., 2011; Ito et al., 2013; Pavesi et al., 2013; Baldelli et al., 2014; James et al., 2015). Typically, excessive nNOS activity leads to the overproduction of nitrogen oxides, which poses a risk of neurotoxicity following acute brain injury (Yu et al., 2008; Zhou et al., 2010; Yin et al., 2013; Favié et al., 2018; Qu et al., 2020). Therefore, the downregulation of nNOS has significant neuroprotective potential. However, conventional “direct” nNOS inhibitors that target the active site of the enzyme may have adverse effects, given the physiological presence of nNOS at certain concentrations and the poor specificity of these inhibitors (Poulos and Li, 2017). Based on our findings, we can pursue the development of specific mutations in the nNOS-CaMBD peptide that selectively interfere with the SUMOylation hyperactivation mechanism of nNOS. This approach holds promise for the treatment of diseases associated with excessive nNOS activity. However, challenges remain, particularly in comprehensively understanding the competitive interplay between CaM allosteric activation and SUMOylation.

In summary, our research outcomes not only establish a theoretical framework for understanding CaM-specific binding within the CaMBD region of nNOS under non-bonding forces, but also highlight key amino acid residues within the nNOS-CaMBD region. This structural insight offers a new perspective on CaM-mediated nNOS activation and presents opportunities for designing drugs that specifically target nNOS inhibition.

## Methods

### Model construction

The initial models were constructed using the X-ray structure of CaM in complex with an eNOS-interfering peptide (PDB code: 2LL7). Subsequently, eNOS was removed from the structure. Using Coot (Emsley and Cowtan, 2004), we substituted residues in CaMBD based on the nNOS sequence of rats. Molecular docking studies were conducted using the initial model provided by AudoDock Vina (version 1.2.0) (Trott and Olson, 2010; Eberhardt et al., 2021). Residues identified as flexible, particularly those within the peptide/CaMBD interaction sites, were selected for docking simulations. The potential energy of the system was minimized, and docking conformations were generated using a genetic algorithm. A total number of 100 potential conformations were exported for cluster analysis, focusing on those with the highest docking scores. Representative conformations were confirmed to be consistent with the existing PDB structure of the CaMBD from eNOS bound to calmodulin (PDB: 1NIW).

### Molecular dynamics simulations

All molecular dynamics simulations were conducted using the AMBER 24 program (Case et al., 2024), employing the amber19SB all-atom force field parameters (Tian et al., 2020). To maintain an ionic strength of 100 mmol/L, approximately 52 Na^+^ and Cl^−^ ions were added, and each system was solvated using the OPC water potential within a water box, ensuring a minimum solute-wall distance of 12 Å. To determine the parameters for Ca^2+^ binding to the aspartic acid residue in CaM, B3LYP calculations were performed using the 6-31G* basis set in the Gaussian 16 program (Petersson et al., 1988; Petersson and Al-Laham, 1991; Stephens et al., 1994; Rassolov et al., 2001; Frisch et al., 2016). Parameters of Ca^2+^ binding to relative residues were calculated by VFFDT program (Zheng et al., 2016). The simulation procedures were standardized across all the systems. Initially, the potential energy of each system was minimized to eliminate unfavorable interactions. This was achieved through four rounds of minimization, totaling 2,500 steps. The first two rounds utilized the steepest descent (SD) and conjugate gradient (CG) methods, respectively, with the entire system restrained, except for water molecules and ions. Subsequently, the system was allowed to relax without restraint during the last two rounds. Non-bonded interactions were truncated at 12 Å, and the SHAKE algorithm was employed to constrain hydrogen-containing bonds. Following minimization, the system was heated from 0 K to 300 K over 200 ps under constant pressure (1 bar), with the protein atom positions restrained at a force constant of 10 kcal/(mol × Å^2^). A time step of 2 fs was utilized during this heating phase. Finally, conventional molecular dynamics simulations were performed for 500 ns without restraints. Similar simulation protocols were applied to the other systems. Free energy calculations were performed using the MMPBSA.py script in AmberTools (version 24), employing the MM-GBSA implicit solvent model while maintaining a fixed ionic strength of 100 mmol/L. Other analyses, including polar contact, hydrogen bond, and correlation analyses, were performed using the cpptraj module.

### Cellular NO content experiments

The neurons used in the cell experiments were derived from primary mouse brain tissue, isolated and purchased from Sangon Bio. Neurons were cultured in Pricella’s CM-ZY003 complete neuron culture medium. When the cells reached over 80% confluence, they were passaged. Initially, the cell culture supernatant was discarded, and the cells were washed with 2 mL of PBS and subsequently removed. Following this, 700 μL of 0.25% trypsin was added, and the cells were placed in a CO_2_ incubator for approximately 1.5 min. The cells were observed under a microscope and noted to adopt a round shape, and the culture flask was gently tapped to detach the cells. Subsequently, 2 mL of complete neuronal cell culture medium was added to detach all cells. The cell suspension was transferred to a sterile 15 mL centrifuge tube and centrifuged at 1000 rpm for 3 min at room temperature. The supernatant was discarded, and the cell pellet was resuspended in 1 mL of complete culture medium. Subsequently, 10 μL of cell suspension was mixed with 1 μL of trypan blue, and cells were counted using a hemocytometer. Based on the cell counts, we confirmed that the cells were in the logarithmic growth phase. The cell suspension was evenly distributed in the cell culture dishes. For the experimental groups that required peptide addition, the final concentration of the peptides was standardized to 3.3 μM. The respective peptides were dissolved in culture medium and added to the cells. All experimental and control groups were cultured for 12–24 h to ensure sufficient NO production. The NO content was detected using a nitric oxide content detection kit (Solarbio BC1475) (Li et al., 2022). Other experimental details are provided in the Supplementary materials.

## Data Availability

The original contributions presented in the study are included in the article/Supplementary material, further inquiries can be directed to the corresponding author.
