# Supplementary material for: Polarized benzene rings can promote the interaction between CaM and the CaMBD region of nNOS
Source: Front Mol Neurosci. 2024 Sep 3;17:1461272. doi: 10.3389/fnmol.2024.1461272 (PMC11405375; doi:10.3389/fnmol.2024.1461272)
Supplement: Supplementary file 2 [file Table_1.DOCX]

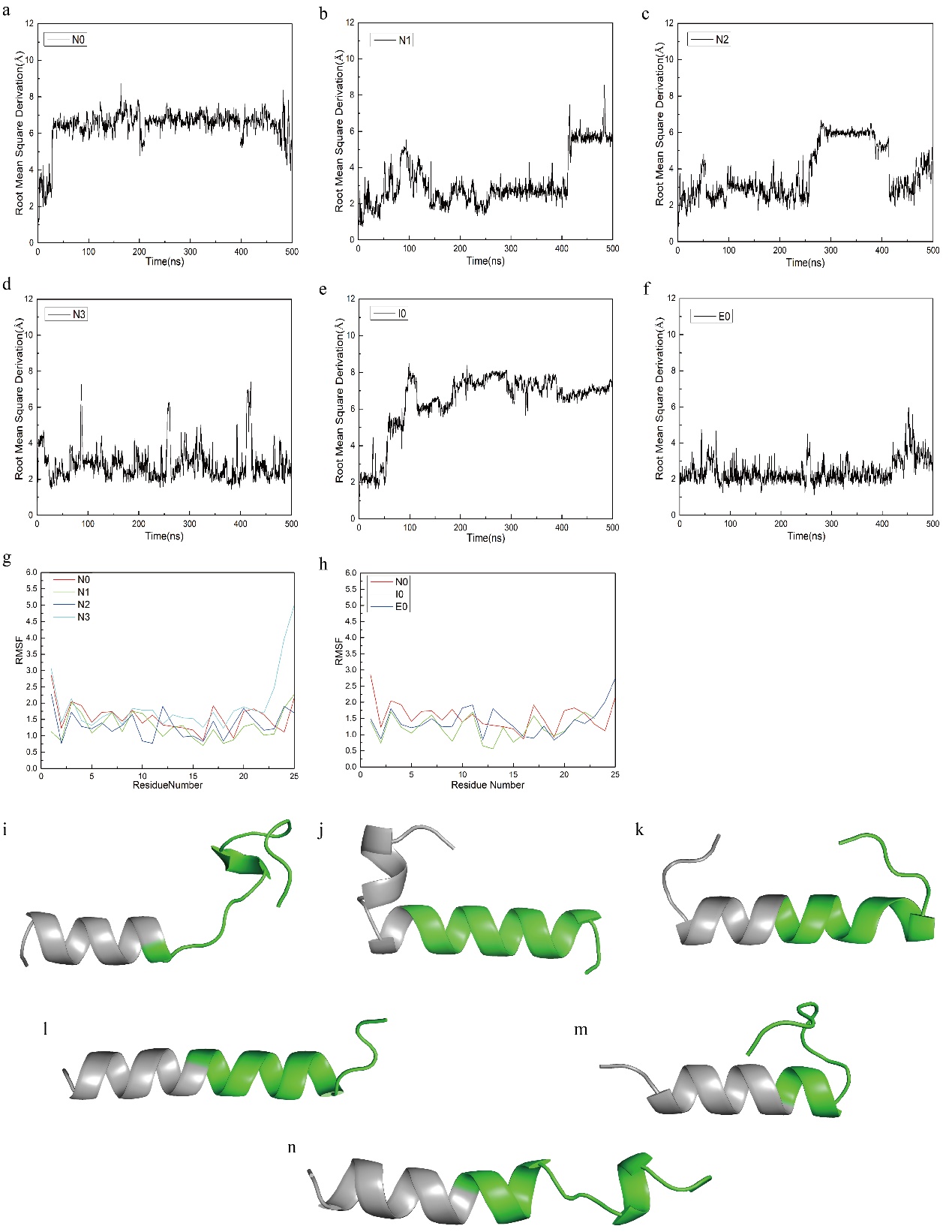


**Figure S1**. Transmembrane peptide sequences (YGRKKQRRR) were added during the simulation of interfering peptides to facilitate their entry into cells. (a-f) RMSD curves of interfering peptides N0, N1, N2, N3, I0, and E0, showing that all peptides reach equilibrium conformation by the end of the simulation. RMSD values remain stable within the normal range, with no abnormalities detected, indicating no structural conflicts. (g-h) RMSF values for each residue of the six interfering peptides, demonstrating similar flexibility across the peptides. (i-n) Equilibrium conformations of interfering peptides N0, N1, N2, N3, I0, and E0 at the end of the 500 ns simulation, showing normal equilibrium conformations with no abnormal structures. Gray regions represent transmembrane peptides; green regions represent interfering peptides.


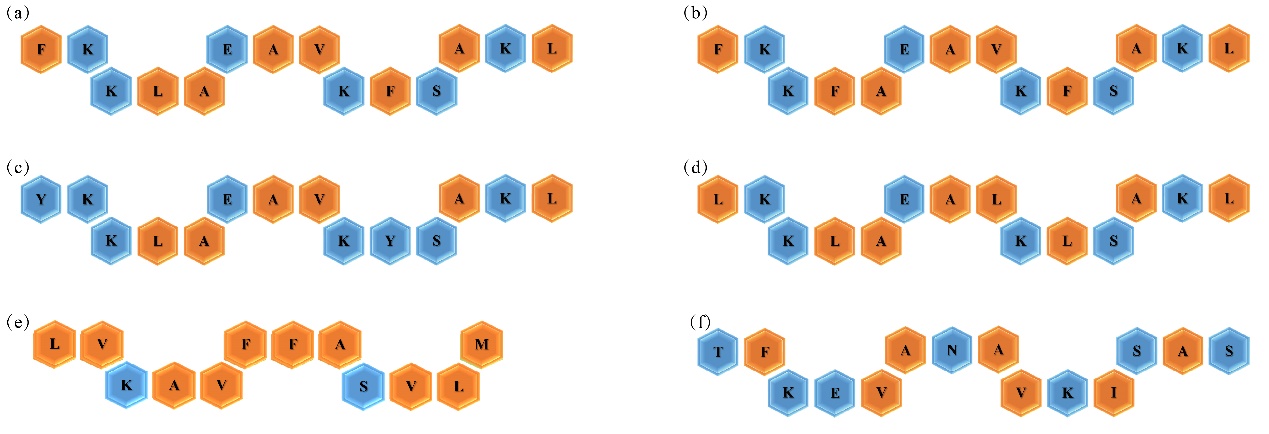


**Figure S2.** (a-f) Amino acid sequences of interfering peptides N0, N1, N2, N3, I0, and E0, respectively. Yellow regions indicate non-polar amino acids, while blue regions indicate polar amino acids.


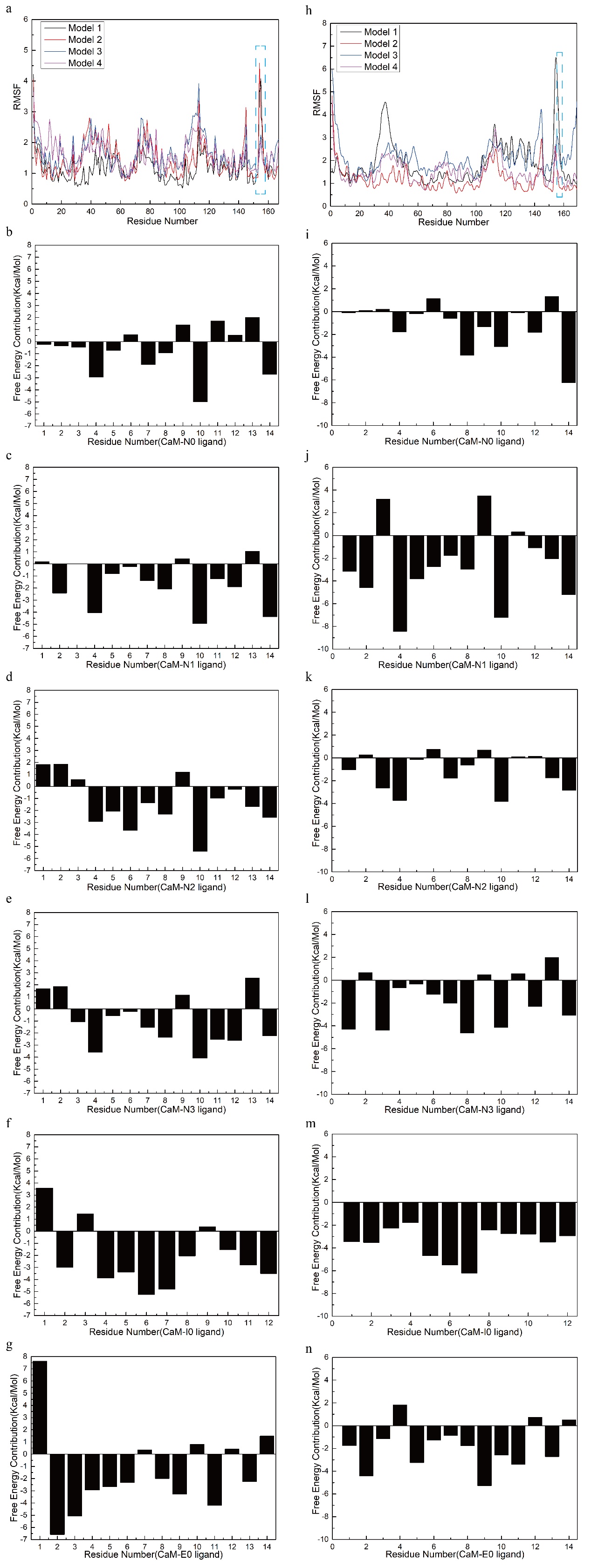


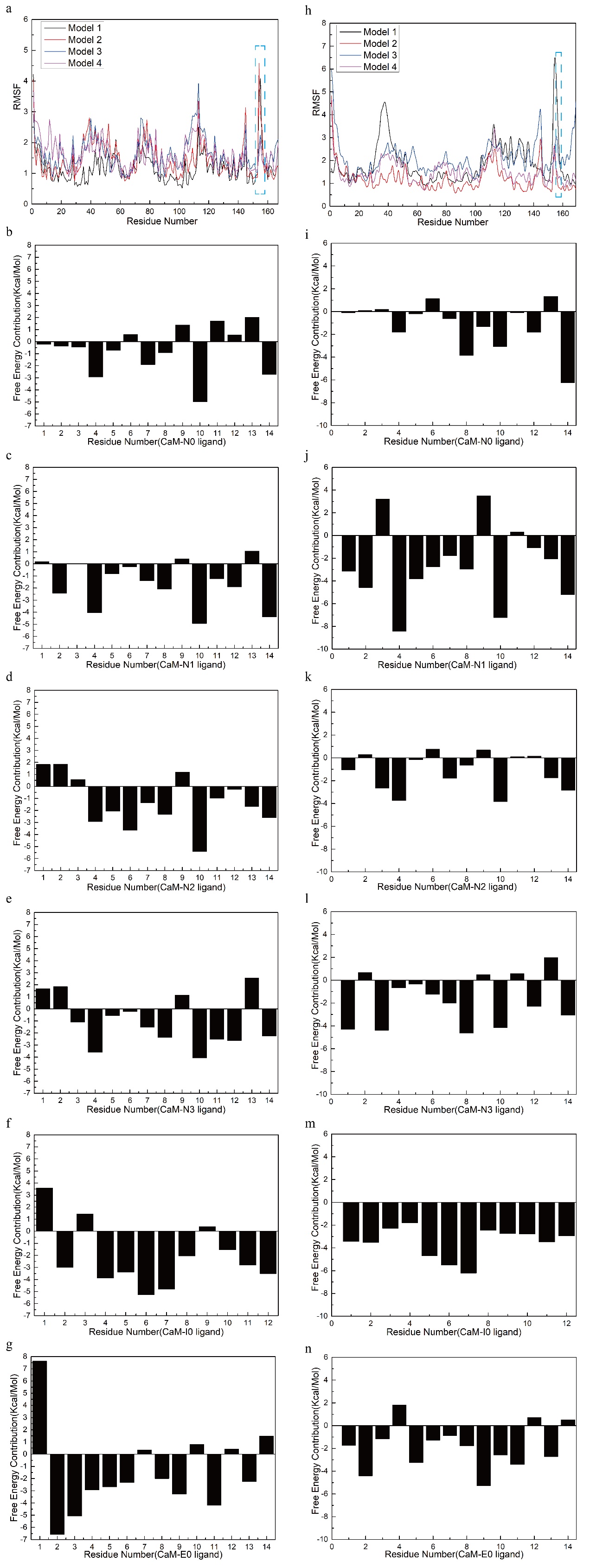


**Figure S3.** (a-g) Simulation data calculated in the TIP3P solvent model. (a) RMSF values for models 1-4, showing that the RMSF values of the N-terminal amino acids of the interfering peptides are higher in the TIP3P model, indicating greater flexibility and more anomalous data compared to the OPC solvent model. (c-g) Free energy distribution data for interfering peptides in models 1-6, showing that the free energy values of the N-terminal and C-terminal amino acids of the interfering peptides are more abnormal in the TIP3P model compared to the OPC solvent model. (h-n) Simulation data for capped interfering peptides calculated in the OPC solvent model. (h) RMSF values for models 1-4, showing that the RMSF values before the blue box are very high, representing the capped amino acids. The RMSF values for the first amino acid of the interfering peptides are normal and lower than those observed in the TIP3P solvent model. (i-n) Free energy distribution data for interfering peptides in models 1-6, indicating that the free energy contributions of certain amino acids are consistent with the results observed in uncapped peptides.

***relevant analytical commands (cpptraj) in molecular simulations:***

***CaM sequence:1-153***

***Interference peptide sequence:154-167***

***①RMSD analysis：***

cpptraj

parm N0.prmtop

trajin N0-mdcrd.nc

strip :WAT

strip :K+

strip :Cl-

trajout N0-mdcrd-vac.nc

run

quit

cpptraj

parm N0.prmtop

parmstrip :WAT

parmstrip :K+

parmstrip :Cl-

parmwrite out N0-vac.prmtop

quit

cpptraj

parm N0-vac.prmtop

trajin N0-mdcrd-vac.nc

rms first @N,CA,C out overall.out

rms first :1-153@N,CA,C out RMSD-CaM.out

rms first :154-167@N,CA,C out RMSD-N0.out

run

***②RMSF analysis：***

cpptraj

parm N0-vac.prmtop

trajin N0-mdcrd-vac.nc 1400 1600(Balanced phase frame)

rms first

rmsf out N0-rmsf.out byres

run

quit

***③relevance analysis：***

cpptraj

parm N0-vac.prmtop

trajin N0-mdcrd-vac.nc 1400 1600(Balanced phase frame)

rms first

matrix correl :* :* byres out correl.out

run

quit

***④hbond analysis：***

cpptraj

parm N0-vac.prmtop

trajin N0-mdcrd-vac.nc 1400 1600(Balanced phase frame)

hbond donormask :1-153 acceptormask :154-167 out hbond1.out avgout hbond-details-1.out

hbond donormask :154-167 acceptormask :1-153 out hbond2.out avgout hbond-details-2.out

run

***⑤Free energy analysis：(MMGBSA)***

cp N0-vac.prmtop complex.prmtop

cpptraj

parm complex.prmtop

trajin N0-mdcrd-vac.nc

rms first

trajout complex.nc

run

quit

cpptraj

parm complex.prmtop

trajin complex.nc

strip :154-167

trajout receptor.nc

run

quit

cpptraj

parm complex.prmtop

trajin complex.nc

strip :1-153

trajout ligand.nc

run

quit

cpptraj

parm complex.prmtop

parmstrip :154-167

parmwrite out receptor.prmtop

quit

cpptraj

parm complex.prmtop

parmstrip :1-153

parmwrite out ligand.prmtop

quit

sbatch mmgbsa.sh

***Cell culture and Immunofluorescence precedures:***

**Primary Neuron Extraction from Mice Brain:**

1. Under sterile conditions, decapitate the mouse and immerse the head in 75% ethanol for 1 minute. Dissect out the intact brain.
2. In pre-cooled dissection solution, remove the meninges and blood vessels, and rinse the brain. Then, use ophthalmic scissors to repeatedly cut the brain into small pieces.
3. Transfer the tissue pieces to a culture dish, remove the dissection solution, and add 2 mL of 0.25% trypsin. Incubate at 37°C for 30 minutes for digestion.
4. Transfer the tissue fragments into a centrifuge tube, discard the remaining digestion solution, and wash the tissue three times with culture medium. After each wash, allow the tissue fragments to settle at the bottom of the tube and discard the supernatant.
5. Finally, resuspend the tissue in 1 mL of culture medium and triturate gently with a Pasteur pipette until the solution becomes turbid. Transfer the supernatant to a culture flask.
6. Add another 1 mL of culture medium and repeat the trituration process 3-4 times until the tissue is mostly digested, discarding the final tissue remnants.
7. Collect approximately 4-5 mL of the supernatant containing single cells into a culture flask.
8. Resuspend the cells in culture medium, count the cells, and seed 1x10⁷ cells into a 6-well culture plate.
9. After 24 hours of incubation, replace the medium with complete culture medium and incubate for 3 days to observe neuronal growth.
10. Replace the medium with cytarabine-containing medium (final concentration 2.5 µg/mL, half-medium replacement) for 3 days to inhibit the growth of glial and non-neuronal cells, obtaining a pure primary neuron culture.
11. Replace the complete culture medium every 3 days, changing half of the medium each time.

**Cell Culture:**

Mouse primary neurons are cultured in complete neuronal culture medium. When the cells reach over 80% confluence, passage the cells. Discard the culture supernatant, wash the cells with 2 mL of PBS, and discard. Add 700 µL of 0.25% trypsin and incubate in a CO2 incubator for approximately 1.5 minutes. Observe under a microscope until the cells become rounded. Gently tap the culture flask to detach the cells. Add 2 mL of complete neuronal culture medium to ensure all cells are detached. Transfer the cell suspension to a sterile 15 mL centrifuge tube and centrifuge at 1000 rpm for 3 minutes at room temperature. Discard the supernatant, resuspend the cells in 1 mL of complete culture medium, and mix 10 µL of the cell suspension with 1 µL of trypan blue for cell counting.

**Immunofluorescence**

1. Cell Preparation: Place a 7 mm × 22 mm coverslip into a culture flask. Add the logarithmic phase cell suspension into the culture flask. Once the cells form a monolayer, remove the coverslip and wash it twice with PBS. For suspended cells, centrifuge and wash twice with PBS, then prepare cell smears.
2. Cell Fixation: Fix the cells with acetic acid/methanol or 95% ethanol for 20-30 minutes.Place the fixed cell coverslip in a staining cylinder, wash with PBS for 5 minutes, and air dry.
3. Add diluted fluorescein-labeled MMP2 antibody and incubate in a humid chamber at 37ºC for 30-60 minutes.
4. Wash twice with PBS for 5 minutes each, followed by one wash with distilled water.
5. Mount the coverslip with 50% buffered glycerol.
6. Observe and photograph the stained specimen promptly.

**Nitric Oxide Content Assay**

NO Content Assay Kit (Solarbio BC1475) was used. Detailed instructions provided in the Solarbio manual were followed in the assay.
